# Femoral locking plate failure salvaged with hexapod circular external fixation: a report of two cases

**DOI:** 10.1007/s11751-016-0254-6

**Published:** 2016-05-27

**Authors:** N. Ferreira, L. C. Marais

**Affiliations:** 1 Department of Orthopaedic Surgery, Tygerberg Hospital, University of Stellenbosch, Cape Town, 7505 South Africa; 2 Tumour, Sepsis and Reconstruction Unit, Department of Orthopaedic Surgery, Greys Hospital, Nelson R. Mandela School of Medicine, University of KwaZulu Natal, Pietermaritzburg, South Africa

**Keywords:** Locking plate, Non-union, Hexapod, Circular external fixator, Reconstruction

## Abstract

Femoral non-unions are difficult to treat even for the experienced orthopaedic trauma surgeon. If the non-union follows failure of modern stable internal fixation, the complexity of the management is further increased. We report two cases of stiff hypertrophic femoral non-unions after failed locking plate fixation that were successfully treated with a new hexapod circular external fixator. In addition to providing the necessary stability for functional rehabilitation and union, the hexapod circular fixator software allows gradual correction of deformities in order to restore the normal mechanical alignment of the limb.

## Background

The use of locking plate technology for orthopaedic trauma has increased in the past 10 years. Their use has a considerable learning curve and is governed by strict biomechanical principles that have to be adhered to [1–3]. Failing to do so can result in a biomechanical environment that is not conducive to fracture healing and may potentially lead to mechanical failure and non-union development [1, 4, 5].

Managing non-unions after internal fixation can be challenging for even the most experienced orthopaedic trauma surgeon [6–10]. There is significant morbidity for the patient in terms of immobility, time away from work, narcotic dependency, and emotional impairment as patients are disillusioned often with medical services [11, 12]. Femoral non-unions in particular have profound influence on quality of life often leading to early retirement and unemployment [13]. The optimal management strategy to promote rapid consolidation of the non-union while simultaneously allowing functional rehabilitation remains unclear.

We report two cases of femoral non-unions associated with failure of locking plate fixation which were successfully treated with the TL-Hex (Orthofix, Verona, Italy) circular external fixator.

## Case 1

A 36-year-old man was referred after failure of internal fixation to an open fracture (Gustilo–Anderson IIIA) of the distal meta-diaphysis of the left femur 5 months earlier. This initial injury was managed by emergency debridement, irrigation and distal femoral locking plate fixation. At presentation with the non-union, the patient had healed scars with no evidence of sepsis. The painful non-union was evident clinically and associated with a varus deformity of the femur in the region of the fracture site.

**Fig. 1:**
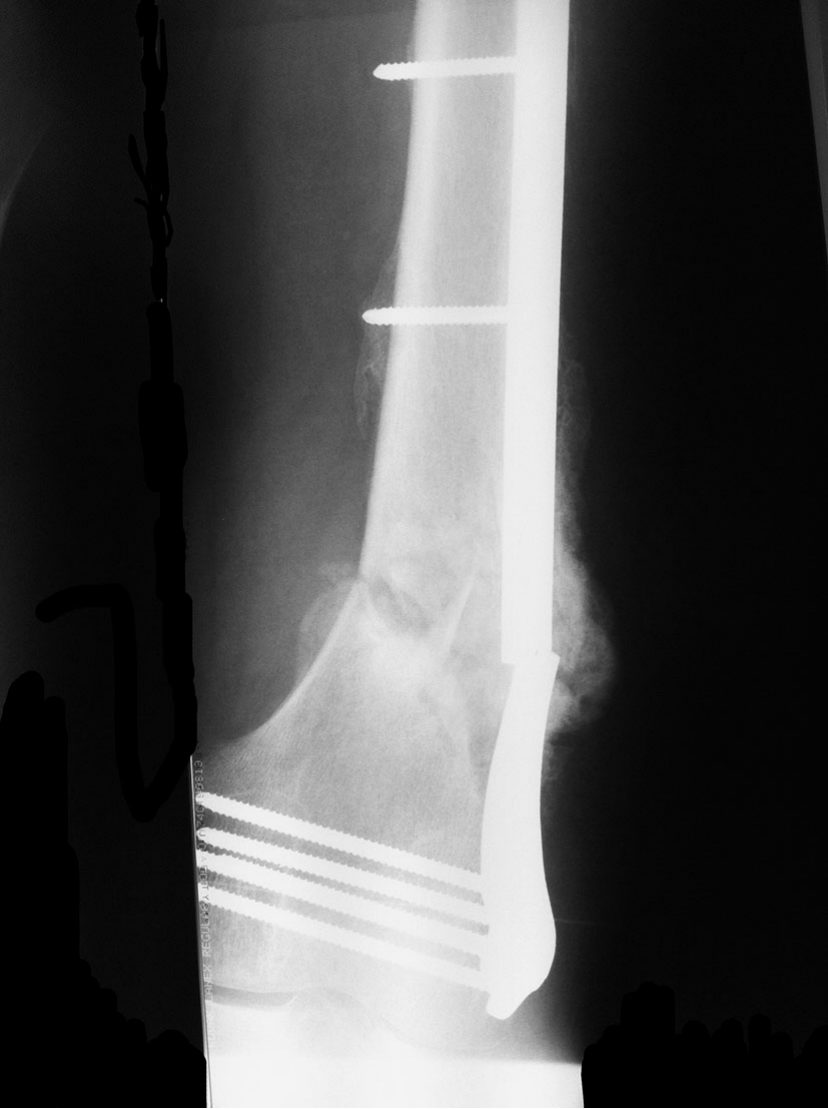
Anteroposterior radiograph of the distal femur demonstrating angulation, nonunion and failed locking plate at the fracture site

Local and systemic staging confirmed the patient to be smoker with no other co-morbidities. Radiographs displayed a broken locking plate and a femoral non-union with a 12° varus and 5° procurvatum deformity (Fig. 1). Knee motion was reduced, with a passive range of motion from full extension to 50° flexion. No evidence of infection was found after routine biochemical investigation and confirmed after intra-operative sampling.

**Fig. 2:**
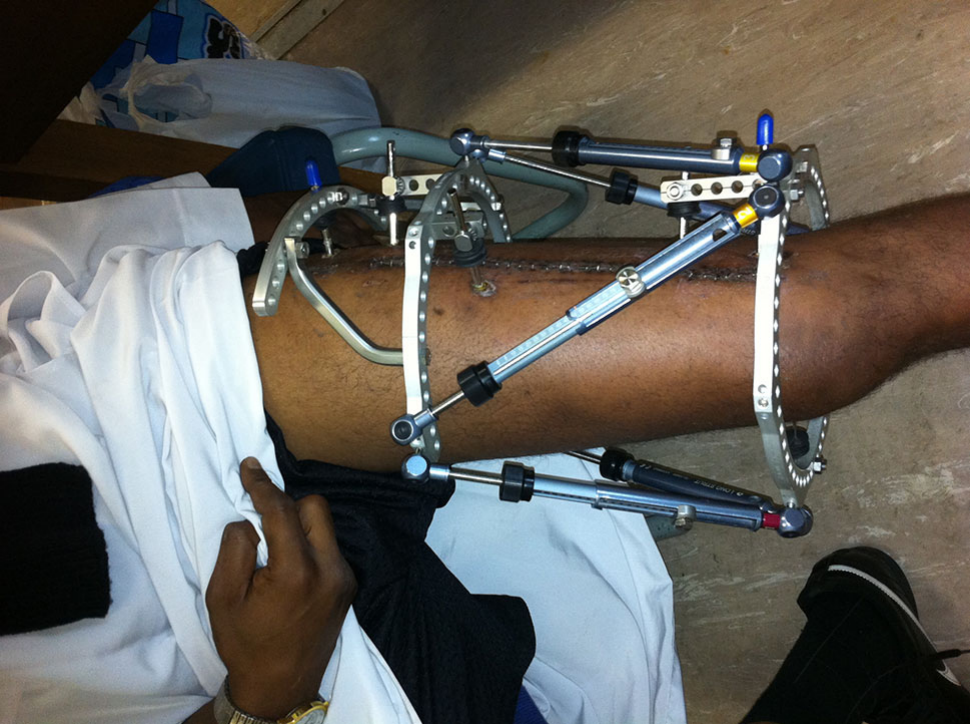
TL-Hex fixator post correction of femoral deformity

Surgery consisted of plate and screw removal through an exposure along the entire length of the plate followed by circular external fixator application (TL-Hex, Orthofix SRL, Verona, Italy) using the ‘rings first’ method. Proximal fixation consisted of three hydroxyapatite coated half pins secured to a 5/8th ring and an arch. Distal fixation consisted of one 1.8 mm tensioned transverse wire and two hydroxyapatite half pins secured to a full ring (Fig. 2). The non-union site was left undisturbed, and no bone graft used.

After a latency period of 7 days, gradual correction was achieved over 6 days. This included 5 mm of distraction at a rate of 1 mm per day to facilitate reduction. Final anatomical alignment in the coronal and sagittal plane was confirmed on radiographs. Functional rehabilitation was encouraged with the assistance of a physiotherapist during the correction and consolidation phases. Full weight bearing was allowed from the first post-operative day. Pin track care followed our standard protocol and included twice daily cleaning with an alcoholic solution of chlorhexidine [14, 15].

The only complications encountered during the treatment period were minor pin track infections. One half pin developed a Checketts and Otterburn stage II infection that responded to oral antibiotics [16]. The tensioned wire developed a stage III infection at a late stage of treatment. The wire was removed without further complications.

**Fig. 3:**
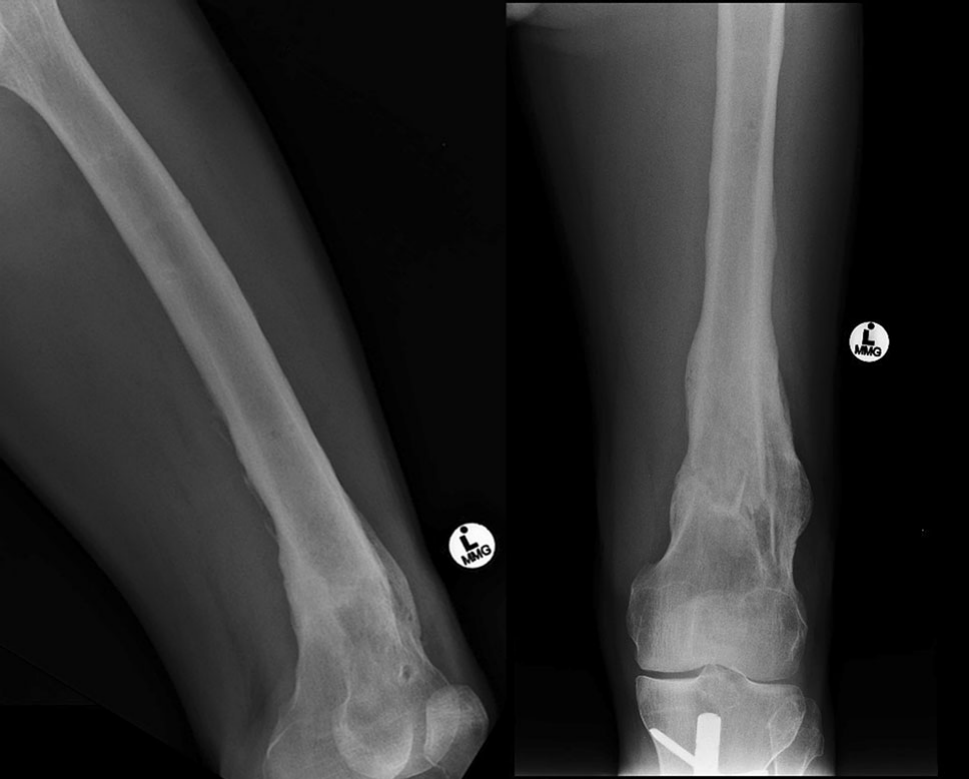
Anteroposterior and lateral radiographs of united femur after hexapod removal

Radiographs confirmed solid union with exuberant callus formation after 13 weeks. The external fixator was removed when painless weight bearing on a dynamized frame was achieved. At last follow-up, 9 months after frame removal, no deformity had occurred at the union site and knee range of motion had improved at full extension to 90° flexion (Fig. 3).

## Case 2

**Fig. 4:**
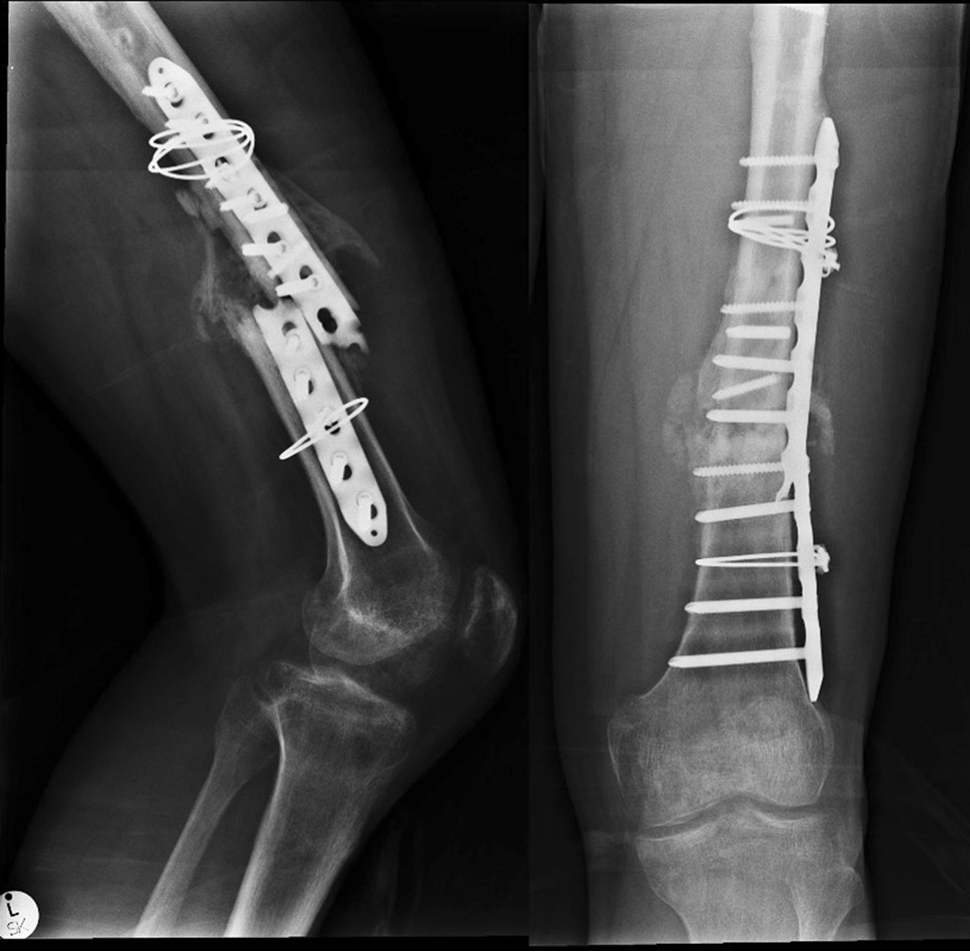
Anteroposterior and lateral radiographs of the femur demonstrating angulation, nonunion and failed locking plate at the fracture site

The second patient had two failed attempts at locking plate fixation of a left femur fracture. This 22-year-old male sustained a closed fracture of the diaphysis treated with a femoral locking plate. After failure at the screw-plate interface, a repeat of the locking plate fixation was performed. This second plate fractured at the femoral non-union site (Fig. 4).

Local and systemic staging confirmed the patient to be a smoker with no other co-morbidities. Radiographs revealed a broken locking plate and a femoral non-union with a 3° valgus, 18 mm posterior translation and 18° procurvatum deformity. Knee motion was reduced, with a passive range of motion from full extension to 70° flexion. Routine biochemical and subsequent intra-operative sampling confirmed no infection.

Surgery consisted of plate removal and circular external fixator (TL-Hex) application. The plate was exposed along its entire length to facilitate removal of all accessible metalware with several broken screws left in situ and the non-union site left undisturbed. External fixation application followed the same design as described in the first case and with no bone graft used.

**Fig. 5:**
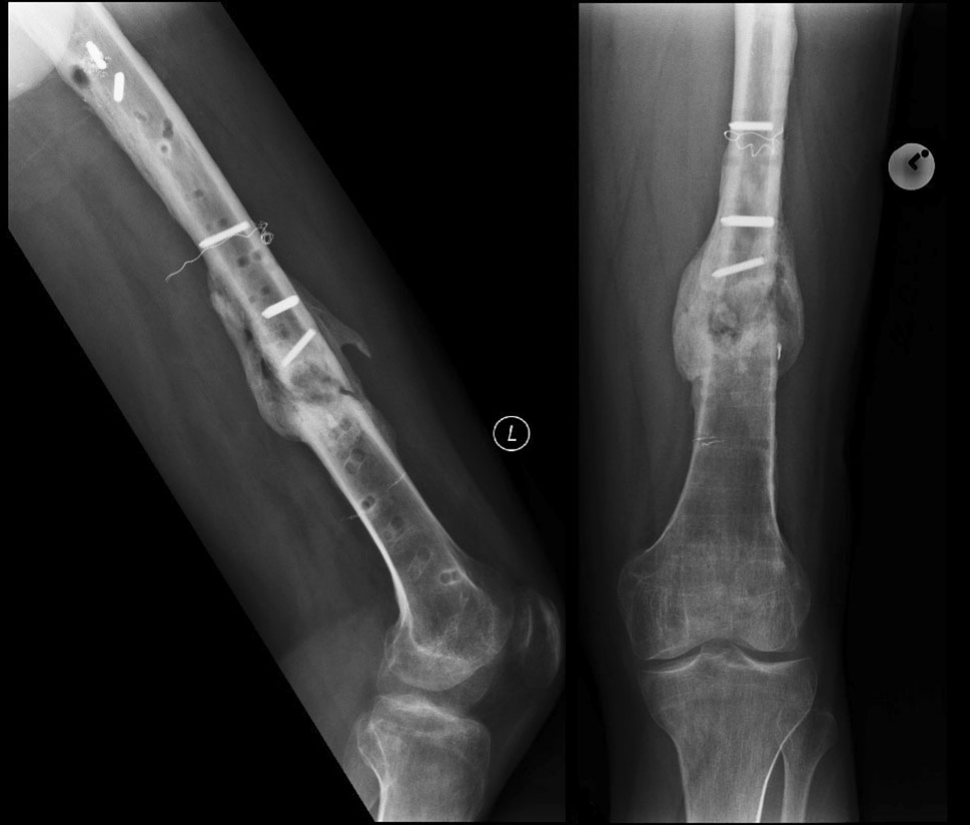
Anteroposterior and lateral radiographs of united femur after hexapod removal

After a latency period of 7 days, gradual correction was achieved over 17 days. This included 5 mm of distraction at a rate of 1 mm per day to facilitate reduction. Final anatomical alignment in the coronal and sagittal plane was confirmed on radiographs. After 14 weeks of functional rehabilitation, solid union was confirmed by radiographs and the external fixator removed. No complications were encountered during the treatment process and at last follow-up, 10 months after frame removal, there was no deformity at the union site and knee range of motion had improved from full extension to 110° flexion (Fig. 5).

## Discussion

Locking plates are fundamentally different from conventional plates [2, 3, 5]. The biomechanical properties of locking plates are, more appropriately, likened to external fixators than traditional plates and screws [5, 17]. Locking plates rely on fixed angle screws to provide stability rather than the friction between the plate and bone generated by screw torque [17]. This intrinsic dissimilarity makes conventional plates and locking plates suited for use in different clinical scenarios [3, 18]. Conventional plates are ideal for achieving union through primary bone healing, with precise reduction, interfragmentary compression and rigid fixation [5, 17, 18]. Locking plates on the other hand are better suited for providing elastic fixation that result in secondary fracture healing with callus formation [3, 5, 17, 18].

When the biomechanical principles of locking plates are not adhered to and these plates are applied like conventional plates, a high strain environment may result that exposes the fracture site to potential non-union formation and construct failure [1, 5, 18]. The human body naturally heals fractures by minimising strain across the fracture site. This is achieved by either decreasing the motion across the fracture site, or by increasing the length of the fracture gap [18]. When there is very rigid fixation, resorption at the fracture site attempts to decrease the strain by increasing the gap length [3, 18]. This is seen where short locking plates are applied with a high screw density as normally done in conventional compression plating. In this setting, non-union formation may result, ultimately leading to construct failure [1]. This was evident in both our cases where non-union development was followed by implant failure.

Non-union in the setting of failed internal fixation is challenging to manage [19]. Firstly, infection must be excluded as the management of an infected non-union is fundamentally different from aseptic non-unions. Secondly, classifying these non-unions according to the traditional Weber and Cech system might not be appropriate. This classification relies on the radiographic appearance of the fracture ends to distinguish between avascular and hypervascular non-unions but fail to take account of previous fixation or adequacy of fixation [19–21]. Wu et al. [19] have suggested a revised protocol to classify femoral non-unions following internal fixation. The authors considered non-unions with stable fixation as avascular and non-unions with unstable fixation as hypervascular. Their proposed protocol underlines a need to take the non-union pathogenesis into account when considering the management strategy. In both these case examples, after plate failure, the unstable situation led to hypervascular non-unions.

Femoral non-unions have no clear evidence-based treatment guidelines. A recent systematic review by Somford et al. [22] has suggested a treatment algorithm for femoral non-unions. They specifically provide treatment recommendations for femoral non-unions that occur after initial internal fixation, suggesting reamed nailing after previous plating and plate fixation after previous intramedullary nailing. This underlines the basic reconstructive principle that when one mode of fixation has failed, another mode of fixation should be considered for the revision surgery.

Gershuni [23] outlined the principles for optimal non-union treatment. This included restitution of bony continuity, correction of alignment in all planes, maintenance and recovery of function and limitation of further complications. Hexapod external fixation can fulfil all these requirements. These devices are a modification of the traditional Ilizarov-type fine wire circular external fixator and are able to provide stable fixation and allow early functional rehabilitation [24, 25]. Hexapod fixators consist of two rings connected with six oblique struts in an octahedral configuration. Mathematical algorithms calculate strut length adjustments in order to manipulate the orientations of the two rings to each other [26, 27]. By attaching each of these rings to a bone segment, their position and orientation can be altered, thereby facilitating the reduction of complex multiplanar deformities.

In stiff non-unions, the ability of the hexapod circular external fixator is to provide controlled correction of existing deformities, but, through gradual distraction, the stimulation of new bone formation. This ‘tension-stress effect’ was initially described by Ilizarov [28–30] and is the biological basis of distraction histogenesis used in limb lengthening and bone transport. It is thus possible, in scenarios involving reduced biological potential, to stimulate natural bone healing without the addition of bone graft or other biologic adjuvants. This was demonstrated in both cases where stiff hypertrophic non-unions healed with exuberant callus formation through gradual distraction without the addition of bone graft.

## Conclusion

Locking plate biomechanics are distinctly different from conventional plating. When locking plate principles are not adhered to, non-unions and fixation failure may result. The salvage for these cases can be difficult as broken metalware, bony destruction and deformity is encountered frequently. This treatment strategy using a hexapod circular external fixator provides the option of gradual reduction of deformities together with stable fixation that allows immediate functional rehabilitation.
